# Effects of 3DVR-haptic simulation training on cognitive functions: a functional neuroimaging pilot study using swLORETA qEEG

**DOI:** 10.3389/fnhum.2025.1709436

**Published:** 2026-01-12

**Authors:** Neshka Manchorova, Dimitar Kolev, Angelina Kirkova-Bogdanova, Nikolay Simeonov, Elena Stavreva

**Affiliations:** 1 Department of Operative Dentistry and Endodontics, Faculty of Dental Medicine, Medical University—Plovdiv, Plovdiv, Bulgaria; 2 Department of Logopaedics, Faculty of Public Health, Health Care and Sports, SWU “Neofit Rilski”, Blagoevgrad, Bulgaria; 3 Department of Medical Informatics, Biostatistics and E-learning, Faculty of Public Health, Medical University—Plovdiv, Plovdiv, Bulgaria

**Keywords:** 3DVR haptic training, cognitive functions, executive function, functional neuroimaging, swLORETA qEEG

## Abstract

**Introduction and aim:**

Technological advancements in neuroscience have transformed our understanding of the processes underlying behavior and cognition. The study aims to investigate the changes in functional brain cognitive networks activated by 3DVR haptic-based simulation training in endodontics.

**Materials and methods:**

Fifteen dental students (7 females and 8 males), mean age 21.5 ± 0.5 years, trained for the first time with a 3DVR-haptic simulator in April 2025, participated in the study. The one-group pretest–postest interventional design was approved by the Ethics Committee of MU–Plovdiv. Before and after a one-hour VR-endodontic access practice, we measured connectivity changes in the 49 Brodmann fields using swLORETA qEEG. Data were standardized using an age-stratified normative base through *Z*-score calculations. The qEEG recordings were processed by Neuroguide 3.3.7 and statistically analyzed with Navistat, *p* < 0.05. To control the cognitive effects the participants completed a classic neuropsychological test for assessing executive functions before and after VR training: the Trail Making Test (TMT) Part A and Part B.

**Results:**

The most significant changes in brain activity were observed in the beta frequency range (18–24 Hz), primarily in the left frontal dorsolateral area, and the left insula and hippocampus. A significant change in activity was found in the mediobasal temporal cortex on both the left and right sides. According to connectomics, the most substantial changes were observed in the structures of 6, 7 and 8 ICN. The TMT data showed significant difference in records of the average time, difference and ratio scores before and after training in VR (*p* < 0.05).

**Conclusion:**

3DVR-haptic simulation training in endodontic actively engages cognitive modalities and brain structers related to executive functions, visuospatial sense, attention, and working memory, resilience to stress, and the need for reward. Objective electrophysiological changes correlate with improvements in neuropsychological performance TMT. Despite the limitations of this study, we argue that swLORETA qEEG method is a novel objective approach with a promising potential for validation the effects of 3DVR-haptic simulation training on cognitive functions, offering better temporal resolution compared to fMRI, along with lower costs and safety.

## 1 Introduction

In recent years, there has been a marked increase in the adoption of augmented reality (AR), virtual reality (VR), and mixed reality (MR) technologies within the education sector. These technologies have proven effective in enhancing learning experiences by improving manual dexterity (Chu et al., 2023; Marsden et al., 2022), psychomotor skills of learners (Dzhendov and Georgieva, 2022; Kaskova et al., 2022). They have also been successful in reducing anxiety and building confidence (Hamza et al., 2023), creating a safe learning environment (Veremeenko et al., 2022), and developing complex clinical competencies (Nurunnabi et al., 2024; Cenzon et al., 2022). Simulations with virtual reality systems provide an immersive experience and a sense of presence and allow for the enhancement of elements of the cognitive process during VR training (Shi, 2024; Lachowicz et al., 2024).

The advantages of VR-based training, compared to training in a conventional environment, include the integration of theory and practice (Chopchik et al., 2023; Hamza et al., 2023), the ability to practice training scenarios repeatedly, and improved outcomes (Hamza et al., 2023; Marsden et al., 2022). They also facilitate analysis of practical task performance (Arutyunov et al., 2023; Gileva et al., 2023), boost self-esteem regarding clinical competence (de la Hoz Calvo et al., 2022), and promote sustained interest and attention (Chen et al., 2024). This approach resonates with the new generation of students, who are more technologically connected. Surveys of dental students’ opinions on the advantages of simulation training in virtual reality show expectations for enhanced motivation for learning, development of critical thinking, confidence, training for decision-making in emergencies, the opportunity to learn from mistakes without risk to the patient (Mouatarif et al., 2024; Dzhendov and Georgieva, 2022; Grancharov, 2023), which alignes with the effects of VR on several cognitive domains: executive functions (improvement in reaction speed, accuracy of execution, concentration, working memory), improved visual–spatial orientation, better resistance to stress factors (Andel et al., 2024; Lachowicz et al., 2023; Kang and Lee, 2023). Immersive VR experiences are associated with increased neuroplasticity, as evidenced by studies that show learning new skills through active gameplay can enhance executive function and form new brain connections (Eng et al., 2020). VR training can modulate neurophysiological pathways critical for learning and adaptation, such as the α5-GABAA receptor, which is involved in synaptic plasticity and memory consolidation (Lawson et al., 2024).

Despite the clear educational benefits of 3DVR technologies, the integration of these technologies into curricula and the educational process remains insufficiently explored. Key questions include the extent to which VR can replace traditional clinical training, whether it can achieve a comparable and stable educational effect, and how to improve training modules to enhance cognitive skills in learners. A significant barrier to progress is the lack of standardized assessment methods (Shi, 2024). Currently, there are no clear benchmarks for validating 3DVR-haptic simulation training in dental education based on precise and objective neurophysiological measures of cognitive effects. Such benchmarks are essential to determine whether these training methods effectively lead to learning and knowledge acquisition.

Most methods for assessing the effects of training in conventional and VR environments are based on the analysis of psychomotor skills, particularly the precision in performing specific applied tasks. While the use of didactic tests in dental education is not a new approach, it often only measures levels of cognitive processing. Tests can reliably assess intellectual achievements, but evaluating psychomotor skills, emotional relationships, and cognitive strategies is much more challenging. On the other hand, assessing cognitive modalities such as the analysis of complex processes, synthesis, associative thinking, logical reasoning, critical thinking, planning, strategy, and problem-solving, as well as dealing with unexpected situations, presents difficulties due to the limitations in detecting each element separately. There are numerous tests with various modifications for cognitive functions. However, these tests often exhibit a high degree of subjectivity, low selectivity, and a lack of discrimination when evaluating specific cognitive modalities. These limitations create a significant research gap in comparing conventional educational methods with technology-based approaches, complicating the validation of new hybrid training methods and the establishment of standards and policies within the educational process.

Objective options to classical neuropsychological tests are the functional neuroimaging methods such as Functional Magnetic Resonance Imaging (fMRI), Magnetoencephalography (MEG), Near-Infrared Spectroscopy (NIRS), and Low-Resolution Electromagnetic Tomography (LORETA qEEG). When combined with simulation training in a virtual environment, these methods can provide comparative data on brain responses (such as density values of the power spectrum, along with the corresponding topographic maps, event and response related potentials) in pre-activity, activity, and post-activity sessions of VR-based cognitive tasks (Mishra et al., 2021). In most clinical studies, fMRI is the preferred method due to its high spatial resolution, which allows for precise localization of cognitive phenomena in the brain (Sevcenko et al., 2022; Xiao et al., 2017; Prochnow et al., 2013). The swLORETA qEEG methodology, applied in the present study, is not inferior to fMRI in terms of localization accuracy but is significantly superior to fMRI regarding temporal resolution. This advantage enables the observation of high-speed cognitive processes that fMRI may not be able to capture. The swLORETA qEEG method is a safe, fast, and accessible option for functional neuroimaging. It provides real-time information about the functional and effective connectivity between neural networks, with standardized results based on age-stratified normative data. The method is successfully used to study cognitive processes. Through this method, Vitkova et al. (2024) revealed that the cognitive process related to the inhibition of memory retrieval involved classical motoric cerebral structures with the left primary motor cortex (M1, BA4), thalamus, and premotor cortex (BA6). Applying swLORETA qEEG, Proverbio and Sanoubari (2024) establish a link between music literacy, enhanced reading skills and the development of a right-sided reading area for notation recognition in musicians.

## 2 Aim

The present study aims to investigate changes in functional brain cognitive networks activated by 3DVR haptic-based simulation training in endodontics using swLORETA qEEG neuroimaging.

## 3 Materials and methods

The study involved fourth- and fifth-year dental students (*n* = 15, 7 women and 8 men), randomly and voluntarily selected. Out of 30 students who were offered participation with information about the objectives and procedure, 23 agreed. Of these, 4 dropped out due to non-compliance with the inclusion criteria, and another 4 gave up after the first measurement. Criteria for inclusion were: voluntary participation, first year dentistry, not trained with a 3DVR-haptic simulator. Exclusion criteria were: history of neurological disease, medication intake in the last 24 h before tests.

The study was conducted in April 2025, and for the participants this is the first training with a 3DVR-haptic simulator.

The one-group pretest–postest interventional experimental design received approval from the Ethics Committee of the Medical University in Plovdiv (Protocol 04/10.04.2025).

The practical training in virtual environment by 3DVR-haptic simulator (VirtEasy Dental, HRV Simulation) included eight endodontic access cavity preparations in lower and upper teeth groups (incisors, canines, premolars and molars) following the pointed contours on respective surfaces. The evaluation of each student was done by recoding surgical time, drilling time, target progress, accuracy (target volume and outside target volume) and scoring awarded by simulator according to the evaluation criteria defined by the department teaching standard.

Before and after a one-hour VR practical session on endodontic access preparation, we collected electroencephalography (EEG) recordings from 19 scalp locations based on the International 10/20 Electrode Placement System. Two EEG recordings, each lasting 5 min, were obtained under two conditions: with eyes closed and with eyes open. The recordings were visually inspected and manually edited to remove any visible artifacts. According to reliability measures, we included only segments with a reliability rate greater than 90% in the spectral analyses. A network filter ranging from 47.5 to 52.5 Hz was applied when necessary. Brain electrophysiological activity values were calculated using the Neuroguide 3.3.7 software program. We followed standard procedures for calculating low-resolution electromagnetic tomography (swLORETA) (Pascual-Marqui et al., 1994). Numerous studies have shown that 19 scalp electrodes are sufficient in number to measure intracranial sources of action potentials, including those from the hippocampus, insula, red nucleus, habenula, and cerebellum (Thatcher et al., 2005).

Talairach atlas coordinates from the average of the Montreal Neurological Institute MRI images (Lancaster et al., 2000) were used and referenced to standard anatomical voxels of 7 × 7 × 7 mm. Voxel groups were defined by clear anatomical landmarks identified by Brodmann in 1909, referred to as Brodmann areas (Fox et al., 1994). Time series of current source vectors in the *x*, *y*, and *z* directions were calculated in the central voxel of each of the 49 Brodmann areas. The activity changes in these areas were aligned with the 18 functional Intrinsic Connectivity Networks (ICNs) identified in BrainMap (Power et al., 2011).

Activity and changes in functional and effective connectivity were monitored across the 49 Brodmann areas in both hemispheres. Electrophysiological markers—absolute power, relative power, coherence, phase slope index (PSI), phase lag, and phase shift (Phase Lock) were studied across all frequency bands: delta, theta, alpha, beta and gamma. Raw data are standardized in *Z*-scores against an age-stratified external reference framework. The resulting *Z*-scores were analyzed with the paired samples *t*-test—before training and after training. The analyses were performed in the specialized software platform NeuroNavigator, created specifically for the swLORETA algorithm for EEG analysis, through the statistical analysis add-on Navistat (Applied Neuroscience, Inc.). Navistat is a powerful statistical tool for comparisons of related and independent samples, fully integrated into NeuroNavigator for detailed and accurate evaluation, interpretation, and analysis of qEEG. The advantage is that the data is obtained, processed statistically and visualized in one software environment, without transferring to other statistical processing software, which guarantees accuracy and selection of appropriate statistical tests. The software presents the results of the analyses from the first and second measurements with *p*-value and standard deviations, marking with a color code the statistically significant differences at the level of significance *α* = 0.05.

To control the cognitive effects of simulation training in a virtual environment on brain structures, as measured by the neuroimaging methodology swLORETA qEEG, before and after the training by 3DVR-haptic simulator participants were administered the classic neuropsychological test for detecting executive functions, the Trail Making Test (TMT A and B). The test consists of two parts: TMT-A, which measures processing speed by having the subject connect numbers in ascending order, and TMT-B, which assesses executive functions like cognitive flexibility and set-shifting by requiring the student to alternate between numbers and letters in sequence. The main metric recorded is the time (in seconds) taken to complete each part, number and type of errors during the task (sequence errors or failure to switch between numbers and letters in TMT-B). The difference scores (TMT-B time minus TMT-A time) and ratio scores (TMT-B divided by TMT-A) were calculated to isolate executive control aspects from general processing speed. The collected scores are interpreted according to normative data adjusted for age and education levels for accuracy. Descriptive statistics for TMT-A and TMT-B by average completion times, errors, difference and ratio scores were performed comparing the results before and after endodontic training by 3DVR-haptic simulator.

## 4 Results

**Figure 1:**
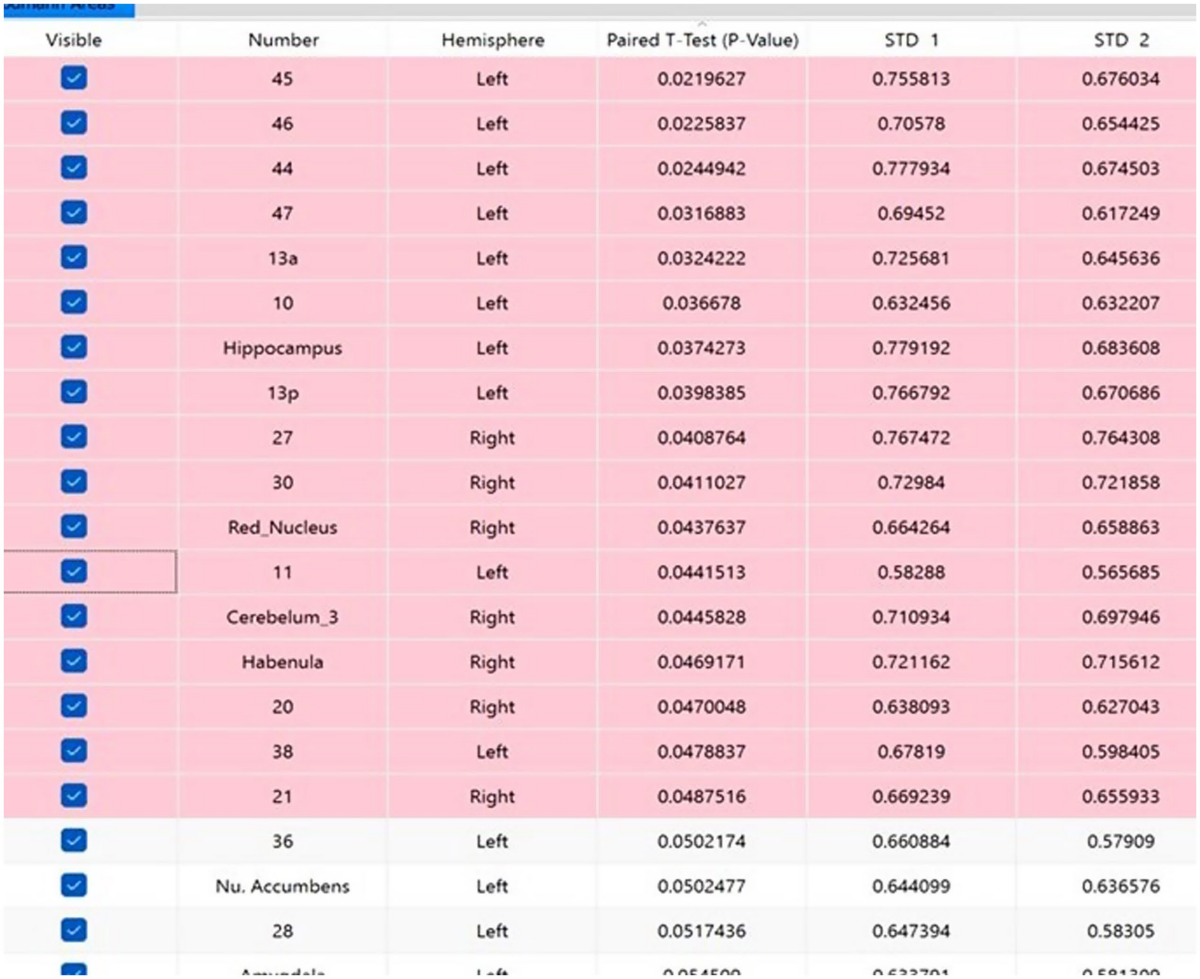
Brodmann areas with a statistically significant change in absolute spectral power in the beta frequency range (18–24 Hz) before and after VR training.

A comparative analysis of the neuroimaging swLORETA qEEG study results reveals a significant increase in both absolute and relative spectral power across multiple brain regions following preparation of an endodontic cavity in a virtual environment using a VR-haptic simulator. The most statistically notable changes in brain activity were found in the beta frequency range (18–24 Hz), predominantly in the left frontal dorsolateral region, which includes Brodmann areas 10 (*p* = 0.037), 44 (*p* = 0.024), 45 (*p* = 0.022), 46 (*p* = 0.023), and 47 (*p* = 0.032), as well as the left insula (*p* = 0.032) and left hippocampus (*p* = 0.037). To a lesser extent, statistically significant changes are observed in the right [Brodmann areas 27 (*p* = 0.041), 30 (*p* = 0.041)] and left [Brodmann area 13p (*p* = 0.039)] medial temporoparietal areas, as well as in the right cerebellum (*p* = 0.045). A statistically significant change in activity was found in the mediobasal temporal cortex on the left [Brodmann areas 38 (*p* = 0.048)] and right [Brodmann area 20 (*p* = 0.047), 21(*p* = 0.049)]. These areas are important brain hubs that actively participate in the neural networks serving executive functions, attention, reward needs, and memory. The observed electrophysiological phenomena indicate the consolidation of neuronal populations in specific brain areas related to certain cognitive tasks. Figure 1 presents the results, as obtained by Navistat, from the Paired *T*-test of *Z*-scores of absolute spectral power in the beta frequency range (18–24 Hz) before and after VR training. Brodmann fields with a statistically significant change are indicated in red. Since the values are standardized to an external reference base, the results before and after VR training are presented by standard deviations STD1 and STD2.

In addition to changes in brain activity within the beta frequency range, statistically significant changes were also observed in the functional and effective connectivity in specific brain regions. These electrophysiological markers reflect the connectivity and functioning of the brain as an aggregate of neural networks. A comparative analysis of the state of neural networks before and after training in a virtual environment using a VR-haptic simulator reveals a statistically significant increase in connectivity and information exchange in brain areas involved in the Executive Function Network, Ventral Attention Network, Emotional Attention Network, and Reward Network.

**Figure 2:**
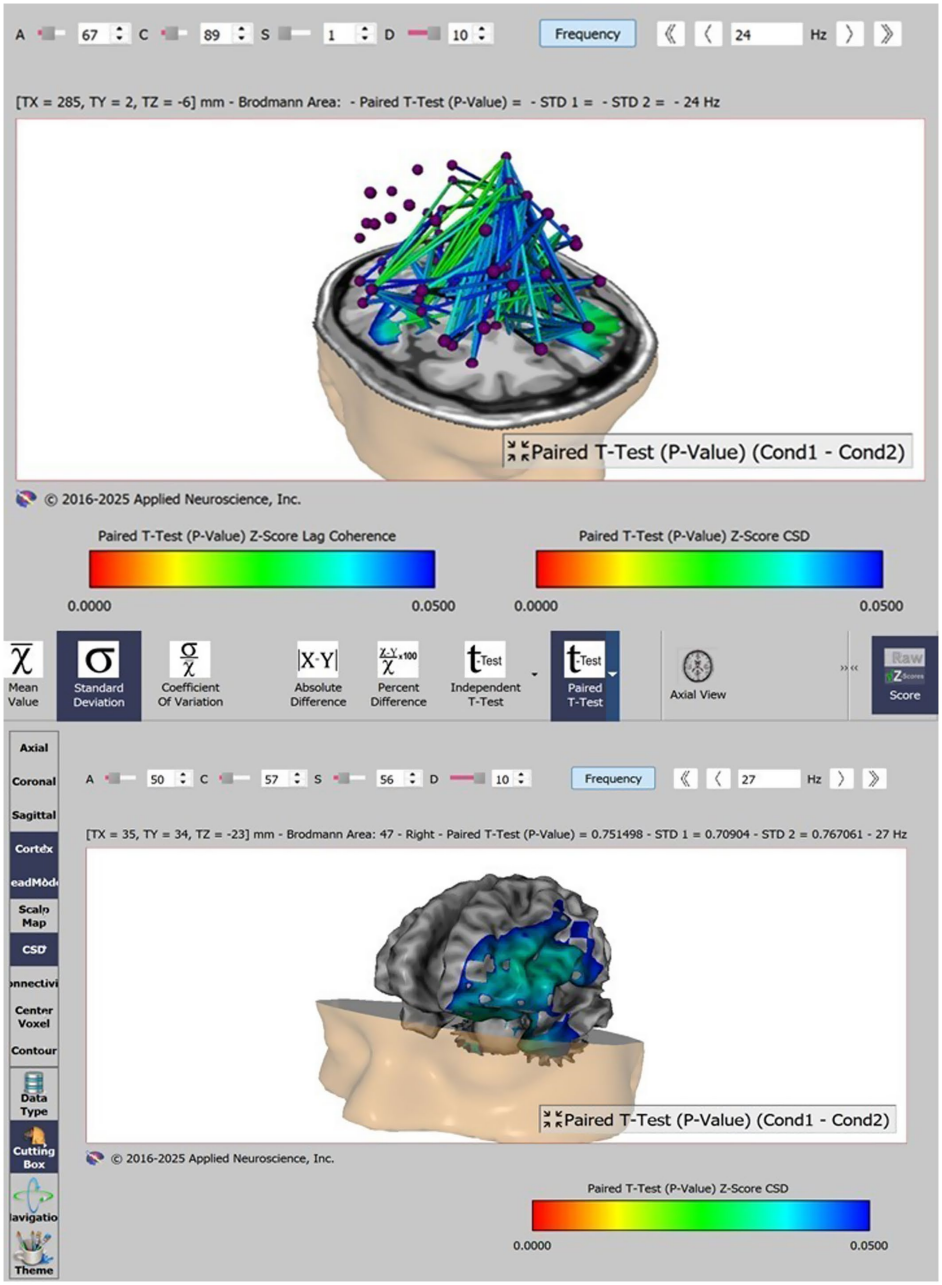
swLORETA qEEG neuroimaging of the regions with statistically significant changes in functional connectivity after VR training.

Figure 2 illustrates the regions with statistically significant changes in functional connectivity after VR training with coordinates: TX 285, TY 2, TZ −6 mm and TX 35, TY 34, TZ −23 mm. The color code represents Paired *T*-test *p* value *Z* score lag coherence and Paired *T*-test *p* value *Z* score CSD (Current Source Density) in the beta frequency dispazone (18–24 Hz), with green links representing *p* < 0.02 and blue links representing *p* < 0.03.

**Figure 3:**
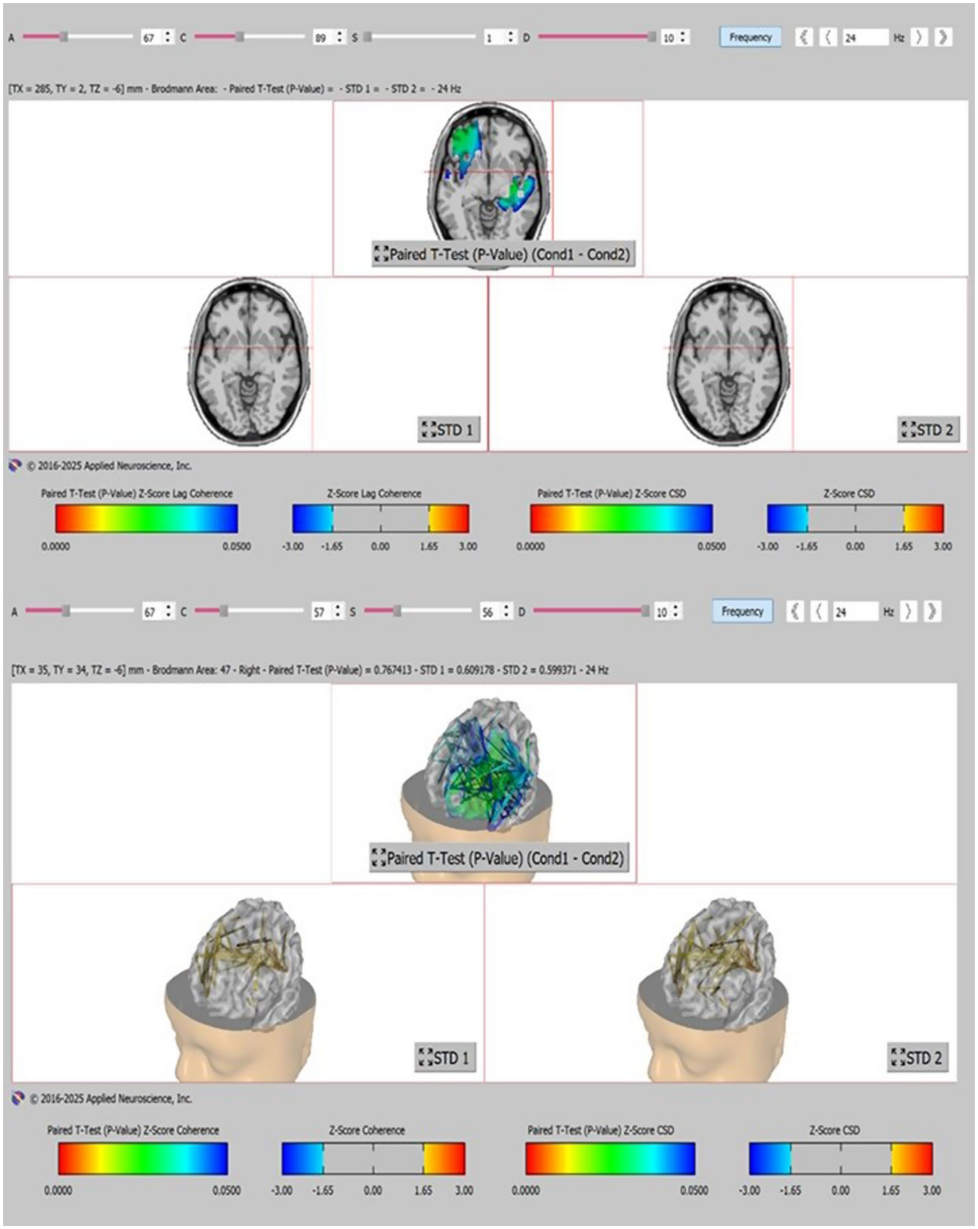
swLORETA qEEG neuroimaging of the regions with significant changes in absolute spectral power in the beta frequency range (18–24 Hz) before and after VR training.

Figure 3 depicts the regions with significant changes in absolute spectral power in the beta frequency range (18–24 Hz) before and after VR training with coordinates: TX 285, TY 2, TZ −6 mm and TX 35, TY 34, TZ −23 mm. The color code indicates Paired *T*-test *Z*-score lag coherence and current source density (CSD). A color code also shows the standard deviations in the interval (−3; 3) of *Z*-scores before VR training (STD1) and after VR training (STD2).

Objective electrophysiological changes correlate with improvements in neuropsychological performance as measured by the applied test for executive functions TMT A, B (Trail Making Test). Participants completed the Trail Making Test Part A in an average of 60.8 s before the VR training and 30.6 after VR training. The average time recorded for completion of Trail Making Test Part B was 95.3 s before the VR training and 71.6 after VR training. Errors were minimal in both records before and after the VR training confirming consistent task switching skills of all participants. Our data showed significant difference in records of the average time before and after training in virtual environment (*p* < 0.05) both in TMT-A and TMT-B tests. The average difference scores (TMT-B time minus TMT-A time) and ratio scores (TMT-B divided by TMT-A) showed improved cognitive flexibility, isolated executive control aspects from general processing speed.

## 5 Discussion

Despite the ongoing use of VR-haptic simulators in various areas of health education in the past decade, the mechanisms through which virtual reality affects functional brain cognitive networks remain unclear. There is a lack of studies on the effects of virtual reality training on different elements in the hierarchy of thinking skills, as outlined in the widely recognized Bloom’s taxonomy of 1956. This taxonomy was revised by Lorin Anderson in 1990 and later by Churches (2008), who introduced new verbs corresponding to the rapid integration of information and communication technology (ICT) in both the classroom and everyday life. In the context of dental education that utilizes VR-haptic simulation, measuring the achievement of specific educational goals relies on assessing the precision of preparation forms and the accuracy in reproducing a specified standard while following a contour. This provides insights into the learner’s manual dexterity and fine motor skills but does fully reflect the attainment of the educational goal as a thinking skill.

The exploration of cognitive modalities is of interest across various fields, including psychology, psychiatry, and pedagogy, often utilizing neuropsychological scales. However, these scales frequently fail to predict cognitive and behavioral functioning in real-world scenarios and are not designed to assess neurobehavioral symptoms (Sbordone, 2014). They are also unreliable for evaluating executive functions and the frontal lobes, which are crucial for planning, decision-making, and social behavior (Sbordone, 2010).

In this context, the mechanistic assessment of the complex behavioral-cognitive characteristics and hierarchical relationships within the thought process in both norm and pathology through neuropsychological scales is one-sided, incomplete. Such assessments may be influenced by various factors, including emotional state, motivation, and environmental influences (Sbordone and Purisch, 1996). Despite these limitations, the use of neuropsychological scales remains a classic and widely accepted approach in the field, backed by long-standing traditions and significant strengths. These scales can be complemented by neuroimaging techniques to validate research findings and test scientific hypotheses.

A comprehensive review of brain activity assessment in immersive virtual reality research during the last decade by Llana et al. (2024) proves that neuroimaging methods integrated with VR spatial-cognitive tasks improve the validity and accuracy of assessment and understanding of cognitive processes.

The study of neural signals and functional brain response through various neuroimaging methods, such as EEG, MEG, MRI, etc., integrated with VR-based stimulation, has significant potential for enhancing our understanding of human perception, memory, cognitive state, dysfunctions, behavior, and other aspects (Mishra et al., 2021). Research has demonstrated that simulation training can influence cognitive load and brain neuroplasticity, as measured by EEG markers, in medical students and trainees performing laparoscopic tasks (Maimon et al., 2022). In recent years, neuroimaging techniques used to assess behavioral and cognitive modalities have advanced significantly. The application of swLORETA qEEG in the present study to evaluate the effect of VR-haptic simulation training on the performance of a practical task in endodontic training of dental students is the first of its kind. The results obtained are innovative, providing an objective registration of stimulated cognitive skills and detailing them by considering each element of cognitive function. A notable advantage of the method is its ability to reveal not only the structural and functional connectivity between individual cognitive neural structures, but also to present the direction and strength of the information flow between the zones as an essential element of their effective communication.

From a neuroanatomical perspective, statistically significantly active BPs in our study are located in the dorsolateral prefrontal cortex (BP 45, 46, 44, 47, 10, 11), in the limbic lobe (BP 13, 27, 30, and hippocampus), and the visual-associative temporopolar cortex (BP 37, 19, and 38).

The dorsolateral prefrontal cortex plays a key role in the implementation of executive functions, managing various cognitive processes such as goal setting, plan building, strategy and decision making; attention and concentration; memory; self-esteem; cognitive flexibility and self-inhibition (Clithero, 2009; Baxter, 2009).

Executive functions encompass high-level thinking skills and decision-making abilities. These functions are represented in four neural networks: the Executive Function Network, the Ventral Attention Network, the Emotional Attention Network, and the Reward Network. The activity of these networks during VR-haptic simulation training illustrates their capacity to achieve learning objectives related to analysis, synthesis, and critical thinking. The executive function network (BP 46, 10, 11) coordinates the abilities to organize, set a goal, and plan action sequences necessary for task completion (motor strategy). These skills are particularly important for dental students in their practical training, as they reflect an understanding of theoretical concepts and a selective choice of appropriate motor skill sequences to achieve their objectives while supporting strategic thinking and decision-making.

Our results demonstrate significant activation of attention and concentration in the field of interest, along with the ability to inhibit distractions. These cognitive elements are critical for maintaining the learning process, especially in the new generation of students who, influenced by technology, tend to multitask but struggle to suppress distractions (Alho et al., 2022; Uncapher et al., 2016). Attention retention often lasts only a few seconds, and the inability to maintain full concentration leads to fatigue and boredom (Hunter and Eastwood, 2016; Danckert and Merrifield, 2016). Training with a VR-haptic simulator allows dental students to significantly enhance their capacity to suppress dominant automatic responses, inhibit negative emotions, and forget intentionally. These results are further supported by the statistically significant activation of the Ventral Attention Network (BP 45, 44, 10, 11, 21), which defines stable attention and concentration based on the value, significance, and importance of the stimulating factor, as perceived by the observer’s beliefs and understandings. The technological approach to training triggers a response in brain structures that are well-trained with means from the familiar and pleasant technological world of the generation (Eng et al., 2020). Integrating educational technology like virtual reality aligns with learners’ expectations for a familiar level of mental stimulation, creating a smoother and less resistant path toward developing high cognitive skills.

Interestingly, our analysis of the neural network associated with attention, known as the Dorsal Attention Network, revealed no statistically significant activation from training with a VR-haptic simulator. This negative result is significant, indicating that brain structures do not perceive virtual reality as an external object. Instead, the brain recognizes VR as part of the learner’s internal experience, without conflict with current reality, spatial orientation, or judgment.

Our results indicate that training in a virtual environment positively impacts brain structures involved in activating working and episodic long-term memory, the ability to understand and reason, which is the basis of learning and cognitive flexibility. We also observed a statistically significant change in electrophysiological markers related to cognitive adaptability, including the ability to shift perspectives, adapt, alter one’s thought processes, as well as self-reflection through self-assessment and self-criticism, and the capacity to understand the emotions of others. Our findings fully support the enhancement of these cognitive skills, as evidenced by a statistically significant activation of the Emotional Attention Network (BP 45, 44, 10, 11, 21).

The diversity of executive functions affected by the virtual learning environment highlights two key aspects. First, it emphasizes the leading integrative role of the prefrontal cortex (Negron-Oyarzo et al., 2024). Second, it illustrates the multi-component effects of learning in a virtual environment that utilizes a haptic-based approach. The involvement of the limbic part of the brain, particularly the retrosplenial cortex, evidenced by a significant activation in Brodmann areas 13, 27, and 30, offers a new perspective on the previously described cognitive effects. These structures in the brain are associated with new cognitive modalities related to self-identification and self-navigation (cognitive and spatial) (Miller et al., 2014). This provides a new insight into the deep mechanisms of learning on the self-awareness of the individual. The functional connection between the limbic lobe and the prefrontal cortex reveals its critical role in learning, motivation, and problem-solving. Brodmann areas 30 and 27 support the brain’s spatial navigation system, consuming up to 40% of the total glucose resource to maintain its metabolic activity (Alexander et al., 2022). This fact underscores their significant evolutionary value. Our results demonstrate statistically significant stimulation, which determines spatial memory and spatial orientation by establishing stable memory traces. The unique role of structures in the retrosplenial cortex is associated with the regulation of two opposing yet interconnected processes: ventral and dorsal attention, internal and external focus (Menon and Uddin, 2010). Achieving a balance between these processes is crucial; it helps avoid attention fatigue and errors of inattention, while also preventing the anxiety that can arise from excessive concentration.

A large volume of publications in the field of dental education investigates stress and anxiety among students (Bakar et al., 2021). Factors that lead to increased anxiety are examined (Lecor et al., 2023), and possible stress-relaxation practices are analyzed (Bakar et al., 2021). However, there is a lack of research on the impact of training methods on dorsal and ventral attention, as well as the regulatory role of the anterior insula, located in Brodmann area 13a, and strategies for preventing the exhaustion of internal attention and overstimulation of external attention to the point of anxiety. Our results present a new perspective on cognitive-behavioral responses in challenging situations. Training in a virtual environment leads to statistically significant activity only in the ventral attention, responsible for concentration, but also has the potential to escalate into anxiety. The obtained results may be explained by the dopaminergic nature of Brodmann area 13, as an integral part of the Reward Network (BP 45, 44, 46, 47, 13, Habenula). In this network, the desire for reward generates arousal, and in situations of overwhelming difficulty associated with insufficient dopamine release, anxiety can emerge (Fakhoury, 2018).

The temporal cortex responds to virtual reality training with statistically significant activation of Brodmann areas 20, 38, and 21. Although these areas are anatomically located in the temporal region of the brain, they also function as part of the limbic system by forming the visual-associative temporopolar cortex. Brodmann areas 38, 20, and 21 work synergistically in visuospatial orientation, contributing to the processing of visual information, visual recognition, visual memory, the integration of various visual elements into overall perception, and the sense of distance (Barense et al., 2011).

The analysis indicated that 3D VR-haptic simulation endodontic training significantly enhances functional connectivity, particularly within the structures of Intrinsic Connective Networks (ICNs) 6, 7, and 8.

ICN 6 is a highly active region in behavioral terms and includes regions of the prefrontal and premotor cortex. It is associated with cognitive visual control of movement, motor timing, and the preparation of the motor act. It is activated during the representation of movement, during the learning and recalling of a complex of movements, and when the gaze is fixed on a specific point.

ICN 7 encompasses the dorsolateral prefrontal cortex (Brodmann areas 44, 45, 46), and the posterior parietal lobe, which are responsible for the visual–spatial processing of information. This network is activated during thinking, calculation, mental rotation, and saccadic eye movements. It is important for attention, adaptive control, and monitoring the performance of a cognitive task.

ICN 8 is a functional neural network that is activated during hand movements, including tapping with fingers, grasping, pointing, electrical, and tactile stimulation in the hands (Laird et al., 2011). These findings are consistent with the expected strong engagement of brain structures responsible for the complex sensory processing of visual, auditory, and haptic stimuli, as well as structures related to executive functions, working memory, and attention, following VR training.

Given the presented data, the limitations in our study are related to the small number of participants, the lack of research on confounding factors, potential for bias and methodological constraints due to the one-group pretest–postest interventional experimental design and lack of comparison to effects of traditional learning methods in clinical environment. Despite these shortcomings, the study is a pilot and for the first time presents the effect of virtual learning environment on cognitive brain structures through functional neuroimaging. The traditional neurophysiological test applied serves as a controlling method for validation of dynamics in executive function parallel to the functional neuroimaging data. Future multidisciplinary research in the fields of behavioral-cognitive neurosciences and educational policies and strategies will enrich our knowledge of building effective personalized models and standards in learning.

## 6 Conclusion

This pilot study represents the first functional neuroimaging investigation into the effects of haptic-based 3D virtual reality (3DVR) training in endodontic education on brain structures associated with specific cognitive functions. These brain regions are crucial hubs actively involved in neural networks that support executive functions, attention, reward processing, and memory.

The incorporation of 3DVR simulation methods as an alternative to traditional training techniques is progressively being integrated into academic programs and curricula. Understanding the structure of tasks and specific time intervals can help measure cognitive load and allow for real-time adaptation of the learning environment (Sevcenko et al., 2022). The impact of these methods on cognitive and behavioral modalities is yet to be investigated in the educational system in depth and with the necessary criticality. The application of functional swLORETA qEEG neuroimaging to objectify cognitive changes in simulated VR training sheds new light on neural network dynamics and illustrates how different training strategies can be evaluated through objective neuroimaging and electrophysiological markers. The method contributes to objective cognitive profiling by allowing for the simultaneous stratification of cognitive elements and their reintegration into a single, indivisible system of cognitive skills, cognitive strategies, behavioral decisions, and emotional judgment.

Future studies will serve as a foundation for the effective implementation of 3DVR-haptic simulators in dental education, in which feedback from the brain’s neural networks will ensure their proper integration with traditional methods. The swLORETA qEEG method, with its non-invasive safe nature and excellent temporal and spatial resolution, is defined as a new objective technique for validating the effects of cognitive training in 3DVR-haptic environments in endodontics.

## Data Availability

The original contributions presented in the study are included in the article/supplementary material, further inquiries can be directed to the corresponding author.
